# The effect of age and clinical circumstances on the outcome of red blood cell transfusion in critically ill patients

**DOI:** 10.1186/s13054-014-0487-z

**Published:** 2014-08-30

**Authors:** Andre Dejam, Brian E Malley, Mengling Feng, Federico Cismondi, Shinhyuk Park, Saira Samani, Zahra Aziz Samani, Duane S Pinto, Leo Anthony Celi

**Affiliations:** Beth Israel Deaconess Medical Center, Boston, MA 02215 USA; Harvard-MIT Division of Health Sciences & Technology, Cambridge, MA 02139 USA; Oakland University William Beaumont School of Medicine, Rochester, MI 48309 USA; Mount Auburn Hospital, Cambridge, MA 02138 USA; Aga Khan University Medical College, Karachi, 74800 Pakistan; Institute for Infocomm Research, Singapore, 138632 Singapore

## Abstract

**Introduction:**

Whether red blood cell (RBC) transfusion is beneficial remains controversial. In both retrospective and prospective evaluations, transfusion has been associated with adverse, neutral, or protective effects. These varying results likely stem from a complex interplay between transfusion, patient characteristics, and clinical context. The objective was to test whether age, comorbidities, and clinical context modulate the effect of transfusion on survival.

**Methods:**

By using the multiparameter intelligent monitoring in intensive care II database (v. 2.6), a retrospective analysis of 9,809 critically ill patients, we evaluated the effect of RBC transfusion on 30-day and 1-year mortality. Propensity score modeling and logistic regression adjusted for known confounding and assessed the independent effect of transfusion on 30-day and 1-year mortality. Sensitivity analysis was performed by using 3,164 transfused and non-transfused pairs, matched according the previously validated propensity model for RBC transfusion.

**Results:**

RBC transfusion did not affect 30-day or 1-year mortality in the overall cohort. Patients younger than 55 years had increased odds of mortality (OR, 1.71; *P* < 0.01) with transfusion. Patients older than 75 years had lower odds of 30-day and 1-year mortality (OR, 0.70; *P* < 0.01) with transfusion. Transfusion was associated with worse outcome among patients undergoing cardiac surgery (OR, 2.1; *P* < 0.01). The propensity-matched population corroborated findings identified by regression adjustment.

**Conclusion:**

A complex relation exists between RBC transfusion and clinical outcome. Our results show that transfusion is associated with improved outcomes in some cohorts and worse outcome in others, depending on comorbidities and patient characteristics. As such, future investigations and clinical decisions evaluating the value of transfusion should account for variations in baseline characteristics and clinical context.

**Electronic supplementary material:**

The online version of this article (doi:10.1186/s13054-014-0487-z) contains supplementary material, which is available to authorized users.

## Introduction

In critically ill patients, anemia is common; about 95% of intensive care unit (ICU) patients have abnormally low hemoglobin levels by ICU day 3 [1]. The transfusion trigger of “30/10” (HCT, <30%; hemoglobin, <10 g/dl) was suggested in a case series of trauma patients as early as 1942. Several clinical trials conducted over the past two decades have shown at least equivalent outcomes when a more-conservative transfusion threshold of hemoglobin 7 g/dl is applied to a critically ill patient population [2–5], whereas other prospective and retrospective studies have shown a hazard associated with RBC transfusion >7 g/dl in a variety of patient populations [3,4,6–12]. This heterogeneity in outcomes suggests that the impact of RBC transfusion on mortality varies depending on the patient population studied, the transfusion threshold used, and the age of the RBCs transfused [13]. Multivariate regression analysis, adjusting for illness severity, reported that RBC transfusion was not associated with an increase in mortality among ICU patients. In fact, a protective effect at 30 days was noted after propensity score matching [9,14,15]. For patients with cardiovascular disease, the relation between RBC transfusion and clinical outcome is even more complex. Data from almost 79,000 patients [16] older than 65 years who had been hospitalized with a diagnosis of acute myocardial infarction (MI) found an association between the RBC transfusion and improved outcomes in elderly patients when admission hematocrit values were <33%. Conversely, other retrospective trials have consistently shown that RBC transfusions are associated with adverse outcome in patients undergoing coronary bypass surgery or acute coronary syndrome [17]. The recent randomized, single-center TRACS (Transfusion Requirements after Cardiac Surgery) trial compared a restrictive with a liberal strategy (transfusion for hematocrit <24% or <30%, respectively), and reported no difference in the composite end point of 30-day mortality and morbidity (cardiogenic shock, acute respiratory distress syndrome, or acute kidney injury) [18].

We hypothesize that these varying results stem from a complex interplay between RBC transfusion, patient characteristics, and clinical context. Specifically, we aim to test whether age, comorbid conditions, and clinical context modulate the effect of RBC transfusion on mortality. We therefore analyzed the impact of RBC transfusion in the critical ill not only in the overall population but also in clinically important subgroups of patients in the Multiparameter Intelligent Monitoring in Intensive Care II (MIMIC-II) database.

## Methods

The MIMIC-II database (v2.6) is a publicly available clinical database developed by the Massachusetts Institute of Technology (MIT), Phillips Healthcare, and Beth Israel Deaconess Medical Center (BIDMC) [19]. MIMIC-II is a repository of de-identified administrative, clinical, and survival outcome data from more than 32,000 critically ill patients treated in the ICUs at BIDMC from 2001 through 2008. These data include clinical variables such as patient age, gender, and chronic disease diagnoses, as represented by International Classification of Diseases (ICD) codes; laboratory data such as hematocrit, serum chemistry, and microbiology; physiological data such as blood pressure and heart rate; markers of treatment intensity such as the utilization of mechanical ventilation, renal-replacement therapy, central venous catheters, vasopressors, and blood transfusion; and survival to and after hospital discharge. All data have been de-identified before being archived in the MIMICII database. Patient data were collected only during the patient’s ICU stay. Survival data were obtained from the Social Security database and were available through 2012. All patients admitted (or health care proxies in case the patient could not give informed consent on admission) to any of the intensive care units at the Beth Israel Deaconess gave written informed consent to allow data collection during their ICU stay. These data are stored in the MIMICII database.

The Institutional Review Board of the Beth Israel Deaconess Medical Center (BIDMC) and Massachusetts Institute of Technology have approved the use of the MIMMICII database by any investigator who fulfills data-user requirements. The database is freely accessible to the research community at large. All research was performed in compliance with the Helsinki Declaration describing ethical principles for medical research involving human subjects. During the study period, only leukodepleted blood was transfused at the hospital where the data were collected. The mean age of RBCs transfused at the hospital is 28 days.

The primary study population consisted of adult patients who were admitted to the medical ICU (MICU), surgical ICU (SICU), coronary care unit (CCU), or cardiac surgery recovery unit (CSRU). Inclusion criteria were the occurrence of a nadir hematocrit between 20% and 30% and age of 18 years or older. The nadir hematocrit was used for non-transfused patients, and the pre-transfusion value, for transfused patients. Trauma patients (identified by ICD-9 code and location in the trauma ICU) were excluded. We excluded trauma patients because some of these patients receive transfusion in the prehospital setting. This information is not currently consistently captured in the database. Patients who had multiple hospital ICU admissions during the study period were excluded to avoid associating management strategies used during multiple admissions over the study period to a single mortality outcome. For example, if a patient died within a year of two separate ICU admissions, and the transfusion strategy differed during these two admissions, it would be difficult to determine whether death were associated with the first or the second admission.

The primary end point for the study was 30-day mortality, and the secondary end point was 1-year mortality.

The relation between RBC transfusion, 30-day mortality, and 1-year mortality was examined by using a logistic regression model with the exposure of interest (RBC transfusion or no transfusion) adjusted for the propensity to receive RBC transfusion. A previously validated propensity score model [14] was used to adjust for the likelihood of a patient receiving a RBC transfusion. The variables used for the propensity model are shown in Table 1. The results of this analysis are subsequently referred to as propensity score-adjusted analysis.

**Table 1:** **Description of propensity score model**

	**Coefficient**	**Odds ratio**	**(95% Confidence i**	**Interval]**	***P*** **> z**
Age, per year	0.01	1.01	1.01	1.02	0.00
Gender (female)	-0.09	0.91	0.83	1.00	0.06
Solid cancer	-0.08	0.92	0.82	1.04	0.19
Hematologic cancer	-0.15	0.86	0.65	1.15	0.32
Cirrhosis	0.47	1.60	1.24	2.07	0.00
Congestive heart failure	-0.18	0.84	0.74	0.94	0.00
Diabetes	0.28	1.32	1.19	1.47	0.00
SAPS I, per point	-0.02	0.98	0.89	1.08	0.67
Sepsis	0.07	1.08	1.06	1.09	0.00
Respiratory disease	0.09	1.10	0.96	1.26	0.19
SOFA expiratory, per point	0.06	1.06	1.02	1.11	0.00
SOFA epatic, per point	0.17	1.18	1.09	1.28	0.00
SOFA ematologic, per point	0.20	1.22	1.15	1.30	0.00
SOFA Renal, per point	-0.10	0.91	0.86	0.96	0.00
SOFA CNS, per point	-0.18	0.84	0.80	0.87	0.00
SOFA Cardiovascular, per point	0.24	1.28	1.21	1.34	0.00
Mechanical ventilation	0.56	1.74	1.54	1.97	0.00
Hemofiltration/Hemodialysis	0.53	1.71	1.32	2.21	0.00
Hematocrit, per percentage	-0.27	0.77	0.75	0.78	0.00
Constant	4.07	58.32	34.63	98.24	0.00

A subcohort with patients matched according to their propensity score was created. A “greedy” matching algorithm that matched patients in a 1:1 ratio based on propensity score with a caliper width of 0.2, without replacement, was used. Within the propensity matched subcohort, subsequent logistic regression was performed to evaluate the effect of RBC transfusion on the primary and secondary end points adjusted for the propensity to receive RBC transfusion.

Results from this analysis are referred to as the matched analysis. A *P* value of <0.05 was significant. The effect of RBC transfusion on 30-day and 1-year mortality evaluated in the prespecified groups is listed in Table 2 for both the initial unmatched and the matched cohorts of patients.

**Table 2:** **Propensity-score analysis results for entire cohort**

	**30-day mortality**			**1-year mortality**		
	**Odds ratio**	**(95% CI)**	***P*** **value**	**Odds ratio**	**(95% CI)**	***P*** **value**
All	0.955	(0.844-1.082)	0.469	1.013	(0.916-1.120)	0.803
Medical	1.022	(0.848-1.232)	0.817	1.035	(0.874-1.225)	0.693
Surgical, noncardiac	0.801	(0.620-1.035)	0.09	0.861	(0.701-1.058)	0.155
Surgical, cardiac	2.11	(1.316-3.385)	**0.002**	1.807	(1.351-2.418)	**<0.001**
Acute cardiac	0.754	(0.542-1.048)	0.093	0.791	(0.607-1.031)	0.083
Nonacute cardiac	1.157	(0.793-1.688)	0.449	1.35	(1.031-1.768)	**0.029**
Cancer	0.985	(0.777-1.249)	0.9	0.949	(0.775-1.162)	0.614
GI bleed	0.889	(0.542-1.459)	0.642	1.296	(0.840-1.998)	0.241
SAPS <16	1.24	(1.015-1.514)	**0.035**	1.143	(0.986-1.326)	0.077
SAPS^3^ 16	0.818	(0.698-0.958)	**0.013**	0.929	(0.811-1.065)	0.291
Age <55 years	1.711	(1.222-2.396)	**0.002**	1.362	(1.041-1.783)	**0.024**
Age 55 to 75 years	1.167	(0.953-1.429)	0.136	1.208	(1.030-1.416)	**0.02**
Age >75 years	0.704	(0.588-0.843)	**<0.001**	0.802	(0.689-0.934)	**0.005**

Bold numbers highlight significant differences.

All statistical analyses were performed by using MATLAB version R2010b (MathWorks, Natick, MA, USA) with additional processing of data in Microsoft Excel 2010 (Microsoft Corporation, Redmond, WA, USA) and in R 2.15.1. Matching was performed by using the MatchIt package. For each model, patients with missing data were excluded.

## Results

**Figure 1:**
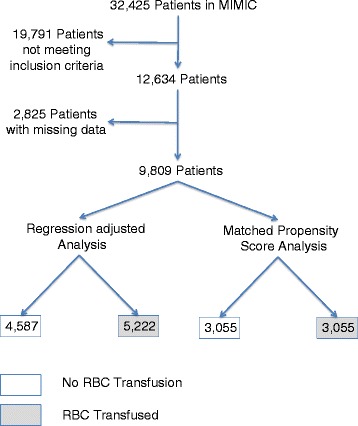
**Description of study design.**

There were 32,425 patients in the MIMIC database, and 12,634 patients met inclusion criteria. Patients were excluded for missing data (*n* = 2,825), leaving 9,809 patients for analysis. Among these, 4,587 patients were given transfusions, and 5,222 patients were not (Figure 1). To assess bias from excluding patients due to missing data, baseline characteristics of excluded patients are shown in Additional file 1 of the supplemental data section (2,513 of the 2,825 total excluded patients; 312 patient did not have patient characteristics available to us). Significant baseline differences were found of the patients included versus those that were excluded for this analysis. The excluded patients had similar 30-day mortality and higher 1-year mortality than the study patient population. SAPS (Simplified Acute Physiology Score) and SOFA (Sequential Organ Failure Assessment) scores were calculated on day of admission. The median SAPS was used to make the variable binary.

The clinical characteristics of the entire cohort are summarized in Table 3. Transfused patients were older, had a lower hematocrit, were sicker, as determined by SAPS and SOFA scores, and had more comorbidities, as defined by the Elixhauser score.

**Table 3:** **Description of entire study cohort**

	**Patients by transfusion status**				
	**All patients**	**Transfused**	**Nontransfused**		
	**(** ***n*** **= 9,809)**	**(** ***n*** **= 4,587)**	**(n = 5,222)**	***P***	***d***
Age, years*	68 (55.7 ~ 78.6)	70.4 (58.3 ~ 79.5)	65.7 (53.5 ~ 77.5)	<0.001	0.239
Female (%)	44.12%	43.73%	44.47%	0.465	0.015
Male (%)	55.88%	56.27%	55.53%	0.465	0.015
Transfused patients (%)	46.76%	100.00%	0.00%	---	---
Nadir hematocrit, percentage*	25.7 (23.6 ~ 27.6)	24.8 (22.9 ~ 26.6)	26.6 (24.5 ~ 28.3)	<0.001	0.657
Nadir hemoglobin, percentage*	8.6 (7.8 ~ 9.3)	8.2 (7.5 ~ 9)	8.9 (8.1 ~ 9.5)	<0.001	
SAPS I score, point	15 (12 ~ 19)	17 (13 ~ 20)	14 (11 ~ 18)	<0.001	0.464
SOFA score, point	7 (4 ~ 9)	8 (5 ~ 11)	6 (3 ~ 8)	<0.001	0.43
Elixhauser Comorbidity, point	2 (1 ~ 4)	2 (1 ~ 4)	2(1 ~ 4)	<0.001	0.086
Medical patients (%)	29.66%	28.28%	30.87%	0.005	0.057
Acute cardiac patients (%)	12.67%	14.45%	11.11%	<0.001	0.1
Surgical patients, noncardiac (%)	24.21%	22.59%	25.64%	<0.001	0.071
Surgical patients, cardiac (%)	33.46%	34.68%	32.38%	0.016	0.049
Nonacute cardiac patients	26.72%	28.45%	25.20%	<0.001	0.073
Hematologic cancer patients (%)	2.58%	2.90%	2.30%	0.061	0.038
Solid cancer patients (%)	16.06%	16.07%	16.05%	0.979	<0.001
Sepsis patients (%)	15.99%	19.03%	13.31%	<0.001	0.156
GI bleed patients (%)	6.41%	10.18%	3.10%	<0.001	0.287

*ks test *P* < 0.0001.

*#median (IRQ) is reported.

The majority of patients received two RBC transfusions during the hospital stay, and most of the RBC transfusion occurred in the first week, 3 days of hospitalization (see Additional file 2 in the supplemental data section). In the overall population, as well as in the matched cohort, no significant association was noted between RBC transfusion and 30-day and 1-year mortality by using propensity-score analysis (see Tables 2 and 4). Timing of transfusion (as a continuous variable) during the ICU stay did not have an impact on 30-day and 1-year mortality (data not shown). RBC transfusion was, however, associated with varying effects on mortality in the subgroups. We first describe the results for the entire cohort and then present the results by using the matched subcohort. In the propensity-score analysis of the entire cohort, transfused patients who were less acutely ill (SAPS <16) as measured by SAPS, had higher 30-day mortality (OR, 1.24; *P* < 0.04; CI, (1.02 to 1.51). Patients with higher SAPS scores (SAPS ≥16) had lower 30-day mortality with RBC transfusion (OR, 0.82; *P* = 0.01; CI, 0.70 to 0.96). Patients undergoing cardiac surgery who were transfused demonstrated the greatest odds of death at 30 days after adjustment for the propensity score (OR, 2.1; *P* < 0.01; CI, 1.32 to 3.39). The increased mortality persisted at 1 year (OR, 1.81; *P* < 0.001; CI, 1.35 to 2.42). Younger patients (age <55 years) had significantly worse outcomes with RBC transfusion at both 30 days (OR, 1.71; *P* < 0.01; CI, 1.22 to 2.40) and at 1 year (OR, 1.36; *P* = 0.03; CI, 1.04 to 1.78). Among patients in the highest tertile of age (age >75 years), RBC transfusion was associated with reduced mortality at 30 days (OR, 0.70; *P* < 0.001; CI, 0.59 to 0.84) and at 1 year (OR, 0.80; *P* < 0.01; 0.69 to 0.93).

**Table 4:** **Results of propensity score-matched cohort**

		**30-day mortality**			**1-year mortality**		
**Studies**	**Cohort size**	**Odds ratio**	**(95% CI)**	***P*** **value**	**Odds ratio**	**(95% CI)**	***P*** **value**
Transfused versus nontransfused	6110	0.93	0.81 ~ 1.07	0.34	0.96	0.85 ~ 1.07	0.45
Medical ICU (MICU)	1792	1.09	0.89 ~ 1.35	0.40	1.16	0.96 ~ 1.4	0.13
Surgical, noncardiac	1360	0.76	0.57 ~ 1.03	0.08	0.80	0.63 ~ 1.02	0.07
Surgical, cardiac	2076	1.83	1.06 ~ 3.17	**0.03**	1.47	1.04 ~ 2.08	**0.030**
Acute cardiac	849	0.77	0.53 ~ 1.12	0.20	0.79	0.59 ~ 1.06	0.12
Nonacute cardiac	1676	1.29	0.82 ~ 2.05	0.27	1.30	0.92 ~ 1.92	0.13
Cancer	1215	1.06	0.81 ~ 1.38	0.69	0.88	0.70 ~ 1.11	0.29
GI bleed	332	0.64	0.34 ~ 1.20	0.17	1.02	0.61 ~ 1.69	0.95
SAPS <15	2441	1.40	1.08 ~ 1.8	**0.01**	1.20	1 ~ 1.46	**0.05**
SAPS ≥15	3657	0.80	0.67 ~ 0.94	**0.01**	0.85	0.74 ~ 0.97	**0.03**
Age <55 years	1308	1.65	1.1 ~ 2.47	**0.01**	1.27	0.92 ~ 1.75	0.15
Age 55 to 75 years	2616	1.21	0.95 ~ 1.54	0.12	1.24	1.03 ~ 1.5	**0.02**
Age >75 years	2152	0.66	0.54 ~ 0.82	**0.0001**	0.73	0.61 ~ 0.87	**0.0001**

Bold numbers highlight significant differences.

After propensity-score matching, 2,167 nontransfused and 1,532 transfused patients were excluded, leaving a matched cohort of 3,055 patients in each group (Figure 1). Propensity-score matching improved the balance of baseline characteristics significantly (see Additional file 3 in supplemental section). Propensity-score matching, however, was not able to achieve a perfect balance between transfused and nontransfused patients. The propensity-score model is well calibrated, as depicted in Additional file 4 in the supplemental data section. After matching, the standardized mean differences, d, for most baseline characteristics between the two treatment groups were less than 10%, indicating a small magnitude of difference.

Nadir hemoglobin was significantly associated with 30-day mortality even after the propensity-score matching, and was added to the regression model in addition to the propensity score. Consistent with the findings in the propensity-score analysis in the initial cohort, propensity-matched patients in the lowest tertile of age had greater odds of 30-day mortality with transfusion (OR, 1.65; *P* = 0.01; CI, 1.10 to 2.47). For patients in the highest tertile of age, transfusion was associated with decreased mortality at 30 days (OR, 0.66; *P* < 0.0001; CI: 0.54-0.80) of age. Again, cardiac surgery patients who were transfused showed much worse outcomes at both 30 days (OR, 1.83; *P* = 0.03; CI, 1.06 to 3.17) and 1-year (OR, 1.47; *P* = 0.03; CI, 1.04 to 2.08).

Evaluation of outcomes among the 629 patients presenting with gastrointestinal bleed (GIB) revealed that three fourths of GIB patients were transfused among patients whose hemoglobin reached a nadir between 7 and 10 g/dl. After adjusting for propensity to transfuse, no significant benefit or harm was found from RBC transfusion in these patients at 30 days (OR, 0.64; *P* = 0.17; CI, 0.34 to 1.2) or at 1 year (OR, 1.02; *P* = 0.95; CI, 0.61 to 1.69).

Bootstrapping of the matched cohort propensity-score analysis by using 1,000 iterations that leave out 50 patients during each iteration validated the findings as listed in Table 4 (data not shown).

## Discussion

Considerable uncertainty remains regarding who might benefit from RBC transfusion and what is the appropriate threshold for transfusion. Our present analysis suggests a complex relation between patient characteristics, clinical context, and outcome. By using regression with adjustment for propensity to transfuse, we observed no significant benefit or harm with RBC transfusions in the entire cohort. However, we observed significant RBC transfusion treatment-effect heterogeneity. In particular, a relation was found between age and outcomes after transfusion. Younger patients derived harm, whereas older patients benefited from transfusion. Moreover, cardiac surgery patients who were transfused consistently had markedly worse short- and long-term mortality.

After the pivotal randomized controlled trial of Hebert *et al*. [5], several prospective and retrospective trials have shown that RBC transfusions are associated with increased mortality in critically ill patients [4], patients with burns [20], cardiac surgery patients [12], and in patients with acute coronary syndromes [6]. Recent trials, however, have challenged the emerging dogma that RBC transfusions are uniformly harmful. Vincent *et al.* [14] performed a retrospective analysis of the SOAP database and found that patients receiving an RBC transfusion had improved survival. The study had a similar analysis and design as the ABC study [4], which showed a clear harm to patients who received RBC transfusions. Vincent and colleagues postulated that the main difference between the ABC and SOAP trials was the proportion of leukocyte-reduced blood used. A later study by Hebert *et al*. [21] also showed that leukocyte-reduced blood improves outcome. We propose that, in addition to leukocyte reduction, patient characteristics and clinical context may play an important role in predicting outcome of RBC transfusion.

The randomized clinical trial of Hebert suggested improved outcome with a conservative approach to transfusion. In the study, the benefit of a conservative strategy was greater among younger patients and those with an APACHE II score <20. The results of our analyses corroborate these findings by showing higher mortality for both younger and less-ill patients who were transfused. We extend Hebert’s observations by showing older and sicker patients who received RBC transfusions had a lower adjusted mortality. This finding has important clinical implications. These data suggest that universal transfusion protocols may be harmful and that the decision to transfuse should include assessment of factors including patient demographics and clinical context. RBC transfusion should not be viewed as uniformly hazardous, and the recent adoption of conservative transfusion practice across the board should be reevaluated.

Moreover, our findings may help reconcile reports describing discrepant clinical outcomes after RBC transfusion on clinical outcome in the literature. For example, the analysis of 79,000 Medicare patients with acute coronary syndrome and a mean age 78 years showed that RBC transfusion was associated with increased survival. The study was contested because it was not consistent with the findings of Hebert *et al*. The discrepancy may be in part attributable to the ages of the Medicare patients [16].

A consensus has emerged to limit RBC transfusion in the absence of evidence of end-organ ischemia. Previously, two exceptions to this rule were GI bleeding and patients with coronary artery disease. A recent randomized controlled study by Villanueva *et al*. [22] showed a survival benefit for patients presenting with active GI bleed when treated with a conservative transfusion strategy. In our analyses of patients with GI bleeding, RBC transfusion did not have an impact on mortality. However, our analysis has to be interpreted with caution because of low numbers of patients who were not transfused, limiting the ability to identify harm or benefit from transfusion. A population with GI bleeding 3 times larger than evaluated in this study would be necessary to identify the effect size (hazard ratio of 0.7), as seen in the prospective trial. However, we can exclude a larger degree of harm or benefit associated with RBC transfusion in patients presenting with GI bleed.

Cardiac surgery patients that were transfused had twice the 30-day and 1-year mortality in both the entire cohort and the matched subcohort. This corroborates prior studies on this patient subset [12,23]. It is unknown why these patients demonstrate higher mortality when transfused. This could be because of confounding that could not be identified in this cohort, such as type of cardiac surgery, redo surgery versus not, emergency versus nonemergency surgery, and so on. Surgery, general anesthesia, and chronic coronary artery disease by itself may not explain worse outcome in patients undergoing cardiac surgery because among noncardiac surgical patients and patients with chronic coronary artery disease, we did not observe worse outcomes with transfusion.

Apart from patients presenting with STEMI [24] and elderly patients [16], RBC transfusion has in the past been associated with harm fairly consistently among those presenting with acute coronary syndrome [17]. The harm from RBC transfusion has been ascribed in previous articles to the aging of blood and loss of function of RBCs during storage [25]. In particular, a worsening of microcirculatory function and nitric oxide bioavailability during storage has been described [26–28]. The finding that RBC transfusions are harmful in younger patients and patients with lower SAPS scores in our study may point toward a balance of beneficial biologic effects, such as optimization of tissue oxygen consumption and harmful effects induced by RBC storage.

Our hypothesis is that in patients who are in a state in which tissue oxygen consumption is independent of oxygen delivery, harmful effects (hemolysis and NO scavenging) outweigh beneficial effects (increase in oxygen delivery) of RBC transfusion. Among patients in which oxygen consumption is dependent on adequate oxygen delivery, however, the beneficial effects of RBC transfusion prevail.

Many authors have stressed the need for another randomized controlled trial evaluating the salutary effects of RBC transfusion in patients with a hematocrit between 21% and 30% in the era of leukocyte-depleted blood transfusions. We propose that such randomized controlled trials should be designed to evaluate the effect in several predefined patient subgroups. Until such data are available, results from the present study and other observational studies suggest that it is time to move away from the one-model-fits-all paradigm. Analysis of large databases is valuable by providing hypotheses to test and, as in our case, propose prespecified subgroup analyses to test in other orthogonally related databases or randomized controlled trials.

As is the case with all retrospective analyses, limitations of this study include exclusion of patients with missing data as well as an inability to identify and correct for unmeasured confounding, as well as to prove causality in the association of RBC transfusion and mortality; the findings should be viewed as hypothesis generating. We attempted to quantify and adjust for known confounding by using modeling techniques and by analyzing baseline characteristics of patients excluded, and we found that excluded and selected patients have similar patient characteristics. We cannot account for selection bias and clustering of outcome because of the single-center nature of the study. Data regarding the indications for blood transfusion and length of storage of RBCs were not available, therefore prohibiting any inference about RBC storage and clinical outcomes.

The practicing physician is still left with using clinical judgment in deciding whether to transfuse critically ill patients. The observed differences in clinical outcome depending on patient characteristics and clinical outcome, as demonstrated in this article, suggests the need for further randomized investigation with predefined subset analyses testing the influence of age and acuity of illness. We need to develop a risk-scoring system for RBC transfusion that estimates patient- and context-specific benefit and risk of harm with RBC transfusion.

## Conclusions

In conclusion, we showed that RBC transfusions in a general intensive care unit population does not increase 30-day and 1-year mortality. However, RBC transfusions have a heterogeneous effect on clinical outcomes in subgroups of this critically ill cohort. Some populations, such as cardiac surgery patients, younger patients, and less-ill patients derive harm, whereas older and more ill patients benefit from RBC transfusion. Additional findings show trends toward worse long-term outcomes among patients with chronic coronary artery disease and improved outcomes among those presenting with acute cardiac disease. Because of the retrospective nature of these investigations, these findings are hypothesis generating rather than showing a clear cause-and-effect relation.

We hope this study encourages the research community to take advantage of large clinical databases in this era of digital health records and use a similar approach of identifying treatment-effect heterogeneity in the evaluation of clinical interventions.

## Key messages

- RBC transfusion does not affect 30-day mortality in a large general critical care population, including surgical as well as medical critically ill patients
- The effect of RBC transfusions differs, however, considerably, depending on patient characteristics and clinical scenario
- Cardiac surgery patient have worse outcomes when transfused
- Older patients as well as patients with a SAPS score of >16 derive benefit, whereas younger (<55 years of age) and less-ill patients (SAPS score <16) derive harm

## Additional files

Additional file 1:
**Comparison of patient demographics of included versus excluded patients (missing data).** GI, gastrointestinal. SAPS, simple acute physiology score. SOFA, sequential organ failure assessment score. Additional file 2:
**Depiction of timing of RBC transfusion (A) and number of RBC transfusions per patient (B).**
Additional file 3:
**Patient characteristics of matched cohort.** *P*, *P* value; d, standardized difference; GI, gastrointestinal; SAPS, Simple Acute Physiology Score; SOFA, Sequential Organ Failure Assessment Score. Additional file 4:
**Calibration of Propensity Score Model.** The propensity-score model is well calibrated in respect to predicting transfusion status and 30-day mortality. Squares represent percentage of 30-day mortality, and diamonds, percentage of patients transfused in respective propensity-score decile.

## Abbreviations

ABC trial: Anemia and Blood Transfusion in Critical Care trial
APACHEII score: Acute Physiology And Chronic Health Evaluation II
BIDMC: Beth Israel Deaconess Medical Center
CCU: cardiac critical care unit
CI: confidence interval
CSRU: cardiac surgery critical care unit
GI: gastrointestinal
GIB: gastrointestinal bleed
HCT: hematocrit
ICD-9: International Classification of Diseases, revision 9
ICU: intensive care unit
MI: myocardial infarction
MICU: medical intensive care unit
MIMICII database: Multiparameter Intelligent Monitoring in Intensive Care II database
OR: odds ratio
RBC: red blood cell
SAPS: Simplified Acute Physiology Score
SICU: surgical intensive care unit
SOAP trial: Sepsis Occurrence in Acutely Ill Patients trial
SOFA: Sequential Organ Failure Assessment score
STEMI: ST-elevation myocardial infarction
TRACS trial: Transfusion Requirement After Cardiac Surgery trial
