# Supplementary material for: The effect of age and clinical circumstances on the outcome of red blood cell transfusion in critically ill patients
Source: Crit Care. 2014 Aug 30;18(4):487. doi: 10.1186/s13054-014-0487-z (PMC4174663; doi:10.1186/s13054-014-0487-z)
Supplement: Additional file 1: — Comparison of patient demographics of included versus excluded patients (missing data). GI, gastrointestinal. SAPS, simple acute physiology score. SOFA, sequential organ failure assessment score. [file 13054_2014_487_MOESM1_ESM.pdf]

Additional File 1: Comparison of Patient Demographics of Study Cohort vs. Patients Excluded Due to Incomplete Data

	Included Patients (n=9809)	Excluded Patients (n=2513)	p-value
Age, years	65.81±0.32	65.43±0.70	<0.0001
Female (%)	44.12%	49.46%	<0.0001
Transfused Patients (%)	46.76%	36.13%	<0.0001
Nadir Hematocrit, percent	25.55±0.05	25.44±0.10	<0.0001
SAPS I Score, point	15.43±0.10	11.55±0.24	<0.0001
SOFA Score, point	6.77±0.07	3.64±0.14	<0.0001
Elixhauser Comorbidity, point	2.47±0.03	2.62±0.07	<0.0001
Medical Patients (%)	29.66%	46.88%	<0.0001
Acute Cardiac Patients (%)	12.67%	14.48%	0.0161
Surgical Patients, Non-Cardiac (%)	24.21%	25.87%	0.09
Surgical Patients, Cardiac (%)	33.46%	12.77%	<0.0001
Non-Acute Cardiac Patients	26.72%	12.85%	<0.0001
Hematologic Cancer Patients (%)	2.58%	4.81%	<0.0001
Sepsis Patients (%)	15.99%	15.44%	0.5
Survivor Length of Stay, days	11.98±0.24	13.32±0.78	<0.0001
30 Day Mortality (%)	15.10%	15.92%	0.31
1 Year Mortality (%)	27.00%	32.51%	<0.0001
