# Supplementary figures and images for: The effect of age and clinical circumstances on the outcome of red blood cell transfusion in critically ill patients
Source: Crit Care. 2014 Aug 30;18(4):487. doi: 10.1186/s13054-014-0487-z (PMC4174663; doi:10.1186/s13054-014-0487-z)

**A**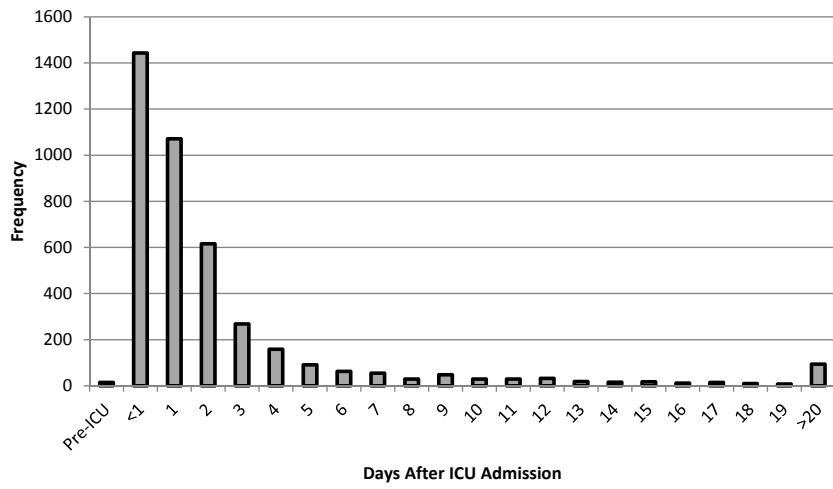**B**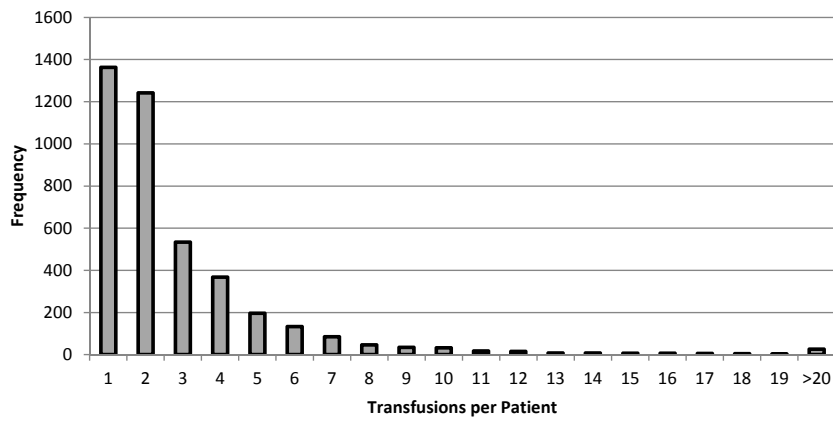

Supplement: Additional file 2: — Depiction of timing of RBC transfusion (A) and number of RBC transfusions per patient (B). [file 13054_2014_487_MOESM2_ESM.pdf]

## Percent Transfused and Percent 30 Day Mortality vs. Deciles of Propensity Score

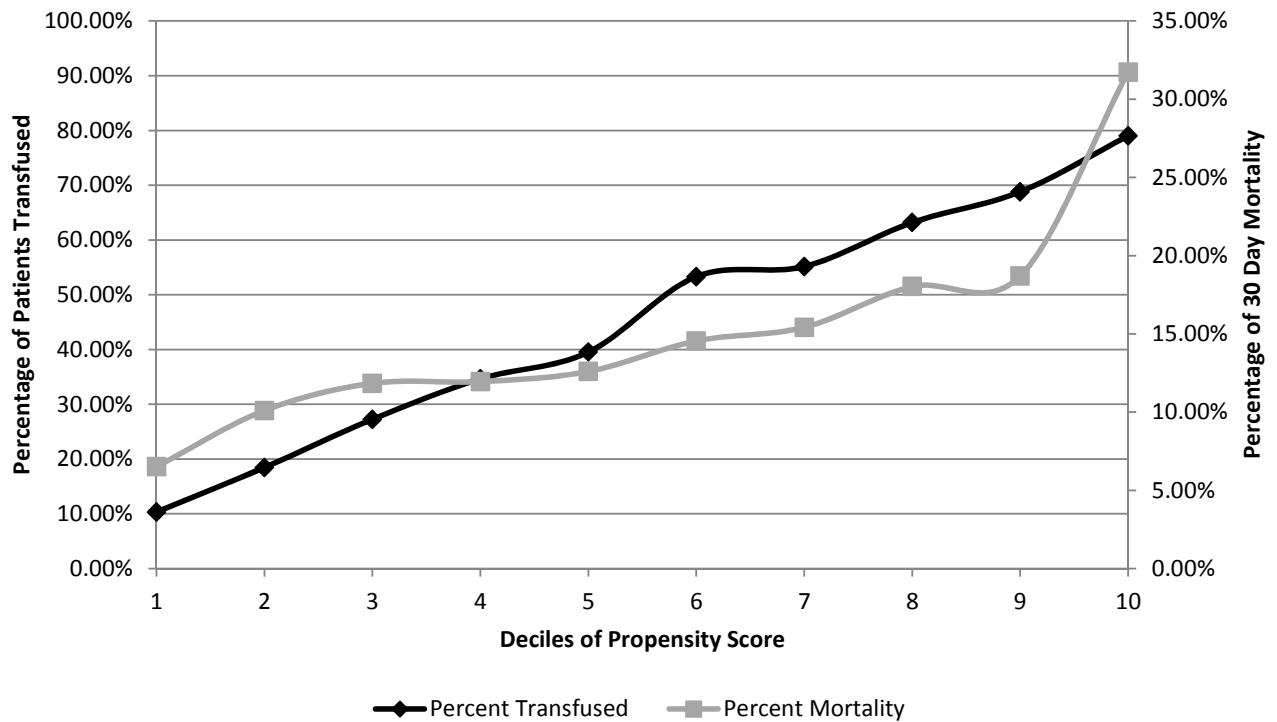

Supplement: Additional file 4: — Calibration of Propensity Score Model. The propensity-score model is well calibrated in respect to predicting transfusion status and 30-day mortality. Squares represent percentage of 30-day mortality, and diamonds, percentage of patients transfused in respective propensity-score decile. [file 13054_2014_487_MOESM4_ESM.pdf]
