# Supplementary material for: The effect of age and clinical circumstances on the outcome of red blood cell transfusion in critically ill patients
Source: Crit Care. 2014 Aug 30;18(4):487. doi: 10.1186/s13054-014-0487-z (PMC4174663; doi:10.1186/s13054-014-0487-z)
Supplement: Additional file 3: — Patient characteristics of matched cohort. P, P value; d, standardized difference; GI, gastrointestinal; SAPS, Simple Acute Physiology Score; SOFA, Sequential Organ Failure Assessment Score. [file 13054_2014_487_MOESM3_ESM.pdf]

Additional File 3: Description of Matched Cohort

	Patients By Transfusion Status				
	All Patients (n=6116)	Transfused (n=3058)	Non-Transfused (n=3058)	p	d
Age, years*	68.7 (57.2~78.7)	68.6 (57~78.4)	68.9 (57.4~79)	0.1	0.02
Female	44.00%	43.00%	44.00%	0.5	0.05
Nadir Hematocrit, percent*	25.4 (23.6~27.1)	25.5 (23.8~27.1)	25.3 (23.4~27.1)	0.01	0.06
Nadir Hemoglobin, percent*	8.5 (7.8~9.1)	8.5 (7.8~9.1)	8.5 (7.7~9.1)	0.8	0.01
SAPS I Score, point*	16 (12~19)	16 (12~19)	16 (12~19)	0.3	0.01
SOFA Score, point*	7 (4~9)	7 (4~9)	7 (4~9)	0.4	0.01
Elixhauser Comorbidity, point*	2 (1~4)	2 (1~3)	2(1~4)	0.001	0.03
**Service unit**				0.37	0.03
Medical Patients	28%	27%	28%		
Acute Cardiac Patients	14%	13%	14%		
Surgical Patients, Non-Cardiac	23%	23%	22%		
Surgical Patients, Cardiac	35%	36%	34%		
Non-Acute Cardiac Patients	28%	30%	27%	0.02	0.07
Hematologic Cancer Patients	2.5%	2.4%	2.6%	0.6	0.02
Solid Cancer Patients	16%	16%	16%	0.6	0.01
Sepsis Patients	16%	15%	16%	0.5	0.05
GI Bleed Patients	6%	9.00%	3.00%	0.0001	0.23

* ks test p-value<0.0001
* median (IRQ) is reported
